# MRI-based clinical radiomics nomogram may predict the early response after concurrent chemoradiotherapy in locally advanced nasopharyngeal carcinoma

**DOI:** 10.3389/fonc.2023.1192953

**Published:** 2023-05-15

**Authors:** Mengxing Wu, Weilin Xu, Yinjiao Fei, Yurong Li, Jinling Yuan, Lei Qiu, Yumeng Zhang, Guanhua Chen, Yu Cheng, Yuandong Cao, Xinchen Sun, Shu Zhou

**Affiliations:** ^1^Department of Radiation Oncology, The First Affiliated Hospital of Nanjing Medical University, Nanjing, Jiangsu, China; ^2^The First School of Clinical Medicine, Nanjing Medical University, Nanjing, Jiangsu, China; ^3^Department of Radiation Center, Shanghai First Maternity and Infant Hospital, Tongji University School of Medicine, Shanghai, China; ^4^Department of Radiotherapy, Nanjing Jinling Hospital, Affiliated Hospital of Medical School, Nanjing University, Nanjing, Jiangsu, China; ^5^Department of Oncology, The Second Hospital of Nanjing, Nanjing, Jiangsu, China

**Keywords:** locally advanced nasopharyngeal carcinoma, radiomics, clinical features, nuclear magnetic resonance, early response and remission, nomogram

## Abstract

**Objective:**

Tumor residue after concurrent chemoradiotherapy (CCRT) in nasopharyngeal carcinoma (NPC) patients often predicts poor prognosis. Thus, the objective of this retrospective study is to develop a nomogram that combines magnetic resonance (MRI) radiomics features and clinical features to predict the early response of locally advanced nasopharyngeal carcinoma (LA-NPC).

**Methods:**

A total of 91 patients with LA-NPC were included in this study. Patients were randomly divided into training and validation cohorts at a ratio of 3:1. Univariate and multivariate analyses were performed on the clinical parameters of the patients to select clinical features to build a clinical model. In the training cohort, the Least Absolute Shrinkage and Selection Operator (LASSO) regression model was used to select radiomics features for construction of a radiomics model. The logistic regression algorithm was then used to combine the clinical features with the radiomics features to construct the clinical radiomics nomogram. Receiver operating characteristic (ROC) curves, calibration curves, and decision curve analysis (DCA) were drawn to compare and verify the predictive performances of the clinical model, radiomics model, and clinical radiomics nomogram.

**Results:**

Platelet lymphocyte ratio (PLR) and nasopharyngeal tumor volume were identified as independent predictors of early response in patients with locally advanced nasopharyngeal carcinoma. A total of 5502 radiomics features were extracted, from which 25 radiomics features were selected to construct the radiomics model. The clinical radiomics nomogram demonstrated the highest AUC in both the training and validation cohorts (training cohort 0.975 vs 0.973 vs 0.713; validation cohort 0.968 vs 0.952 vs 0.706). The calibration curve and DCA indicated good predictive performance for the nomogram.

**Conclusion:**

A clinical radiomics nomogram, which combines clinical features with radiomics features based on MRI, can predict early tumor regression in patients with LA-NPC. The performance of the nomogram is superior to that of either the clinical model or radiomics model alone. Therefore, it can be used to identify patients without CR at an early stage and provide guidance for personalized therapy.

## 1 Introduction

Nasopharyngeal carcinoma (NPC) is a malignant tumor that originates from the mucosal epithelium of the nasopharynx and is typically associated with Epstein-Barr virus (EBV) infection (1, 2). The primary pathological type is non-keratinizing squamous carcinoma (>95%) (3, 4). According to the International Cancer Research Agency, there were approximately 133,000 new cases of NPC worldwide in 2020, accounting for approximately 0.7% of all diagnosed cancers (5). The incidence of NPC exhibits clear regional clustering. In China, it primarily occurs in southern cities such as Guangdong, Guangxi, and Hainan (6). As a consequence of high infiltration, early metastasis and non-specific symptoms of NPC (7), more than 70% of patients present with locally advanced stage (stage III and IV) at time of diagnosis (8).

Currently, concurrent chemoradiotherapy (CCRT) has been the standard treatment for locally advanced nasopharyngeal carcinoma (LA-NPC) (9, 10). Within a few months after CCRT, 58%-97% of patients can achieve clinical complete response (CR) (11–14). However, due to tumor heterogeneity, late clinical stage and high tumor burden, some patients may have residual tumor after treatment. Lee et al. found that early tumor regression predicts better overall survival (OS) and progression-free survival (PFS) in NPC patients (15). Patients with residual tumor have poorer prognostic outcomes compared to those who achieved CR after CCRT (16, 17). Thus, for patients with LA-NPC who have undergone standard treatment but still have residual tumors, timely consolidation or salvage treatments such as surgery, intensive irradiation, or adjuvant chemotherapy are necessary to improve their prognosis. Currently, there is still a lack of efficient and non-invasive tools to early identify people who have difficulty obtaining CR after CCRT.

The rapid advancements in modern medical imaging have made it possible to extract features from tomographic images through high-throughput computing, thereby converting medical images into analyzable data. This process is commonly known as radiomics (18). The main operation steps of radiomics include image acquisition, image segmentation, feature extraction, and model development and validation (19). Due to its high accuracy and availability, radiomics has been extensively researched for its potential in differential diagnosis and prognosis prediction for various types of cancer such as breast cancer, colorectal cancer, esophageal cancer, and other tumors (20–22). However, there are not many studies in the field of nasopharyngeal carcinoma. The aim of this study is to develop and validate an MRI-based clinical radiomics nomogram that can early predict the group of LA-NPC patients who are likely to fail to achieve CR after treatment. This will help to develop individualized treatment for non-CR patients promptly, leading to better survival outcomes.

## 2 Patients and methods

### 2.1 Patients

The data of 145 NPC patients with pathologically confirmed were reviewed and collected in The First Affiliated Hospital of Nanjing Medical University (Jiangsu Province Hospital) from January 2020 to January 2023.The tumor node metastasis (TNM) staging system, as outlined in the 8th edition of the American Joint Committee on Cancer (AJCC), was used to classify the stage of the disease.

This retrospective research enrolled patients who met the following inclusion criteria: 1) pathological confirmation of nasopharyngeal squamous cell carcinoma with III to IVA stage; 2) complete pre-treatment and post-treatment MRI images of the nasopharyngeal neck, including axial T1-weighted images (T1-WI), contrast-enhanced T1-weighted images (T1-C) and T2-weighted images (T2-WI); 3) completion of radical CCRT; 4) available clinical data; 5) adequate bone marrow, liver and renal function. Exclusion criteria included: 1) MRI images with motion artifacts, blurring, or discontinuity; 2) history of prior malignancy or previous treatment for nasopharyngeal carcinoma (NPC); 3) coexistence of immune system diseases or long-term use of hormone drugs.

**Figure 1:**
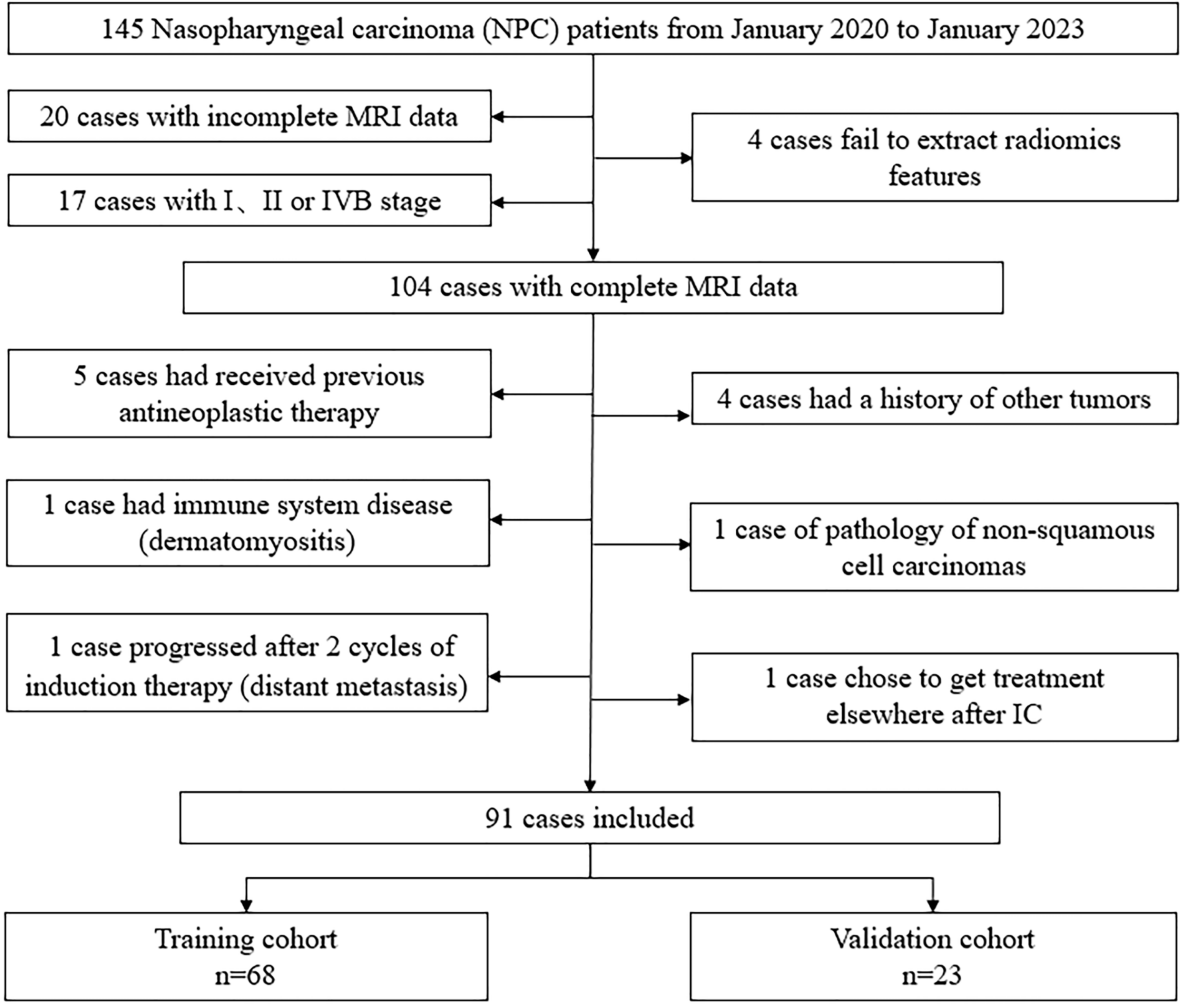
Flow chat of patient selection.

The patient selection process is shown in **Figure 1**. Finally, 91 LA-NPC patients were included in this study.

### 2.2 Treatment

Induction chemotherapy (IC) regimen was taxane and cisplatin (TP): docetaxel 75 mg/m^2^ or paclitaxel 135-175 mg/m^2^ on day 1 and cisplatin or nedaplatin at a dose of 80 mg per square meter on day 1 were administered intravenously once every 3 weeks for 2-3 cycles. CCRT was recommended to be performed within 21 to 28 days after the first day of the last cycle of IC. Radiation therapy (RT) was performed in intensity-modulated radiotherapy mode with 6 MV photon irradiation. The prescribed doses were 66-70 Gy, 64-70 Gy, 60-62 Gy, and 54-56 Gy, in 30-33 fractions, for the PTVs derived from GTVnx, GTVnd, CTV1, and CTV2, respectively. Cisplatin or nedaplatin that was concurrent with radiotherapy was then administered intravenously at a dose of 80 mg per square meter every 3 weeks on days 1, 22, and 43. The quantity of chemotherapy cycles was modified in concordance with the patient’s physical status. It is recommended to undergo an MRI examination within 1-3 months after completing CCRT.

### 2.3 Criteria for tumor response

Tumor response was evaluated by two radiologists, based on MRI images taken before treatment and 1-3 months after completion of CCRT. Using the Response Evaluation Criteria in Solid Tumors 1.1 (RECIST 1.1) (23), patient clinical responses at 1-3 months after CCRT were classified as complete response (CR), partial response (PR), stable disease (SD), or progressive disease (PD). Patients were divided into CR and non-CR groups (PR+SD+PD). Any discrepancies between the two radiologists were resolved through discussion. Cohen’s Kappa value is 0.880, showing that the evaluation is reliable.

### 2.4 Clinical data acquisition

The clinical characteristics before treatment were collected through the health information system (HIS) of Jiangsu Province Hospital. Characteristics included age, sex, height, weight, smoking, drinking, family history, EBV DNA, white blood cell count (WBC), platelet count (PLT), neutrophil count, lymphocyte count, monocyte count, platelet to lymphocyte ratio (PLR), neutrophil to lymphocyte ratio (NLR), lymphocyte to monocyte ratio (LMR), lactate dehydrogenase (LDH), alkaline phosphatase (ALP), serum albumin (Alb), and D-dimer. The volume of nasopharyngeal tumor, maximum coronal length of positive lymph node (N-cor-length), maximal axial diameter of positive lymph node (N-axial-length) and the total volume of lymph node (N-total-volume) were obtained after delineation of the region of interest (ROI) on ITK-SNAP software.

### 2.5 MRI image acquisition, ROI segmentation and extraction of radiomics features

**Figure 2:**
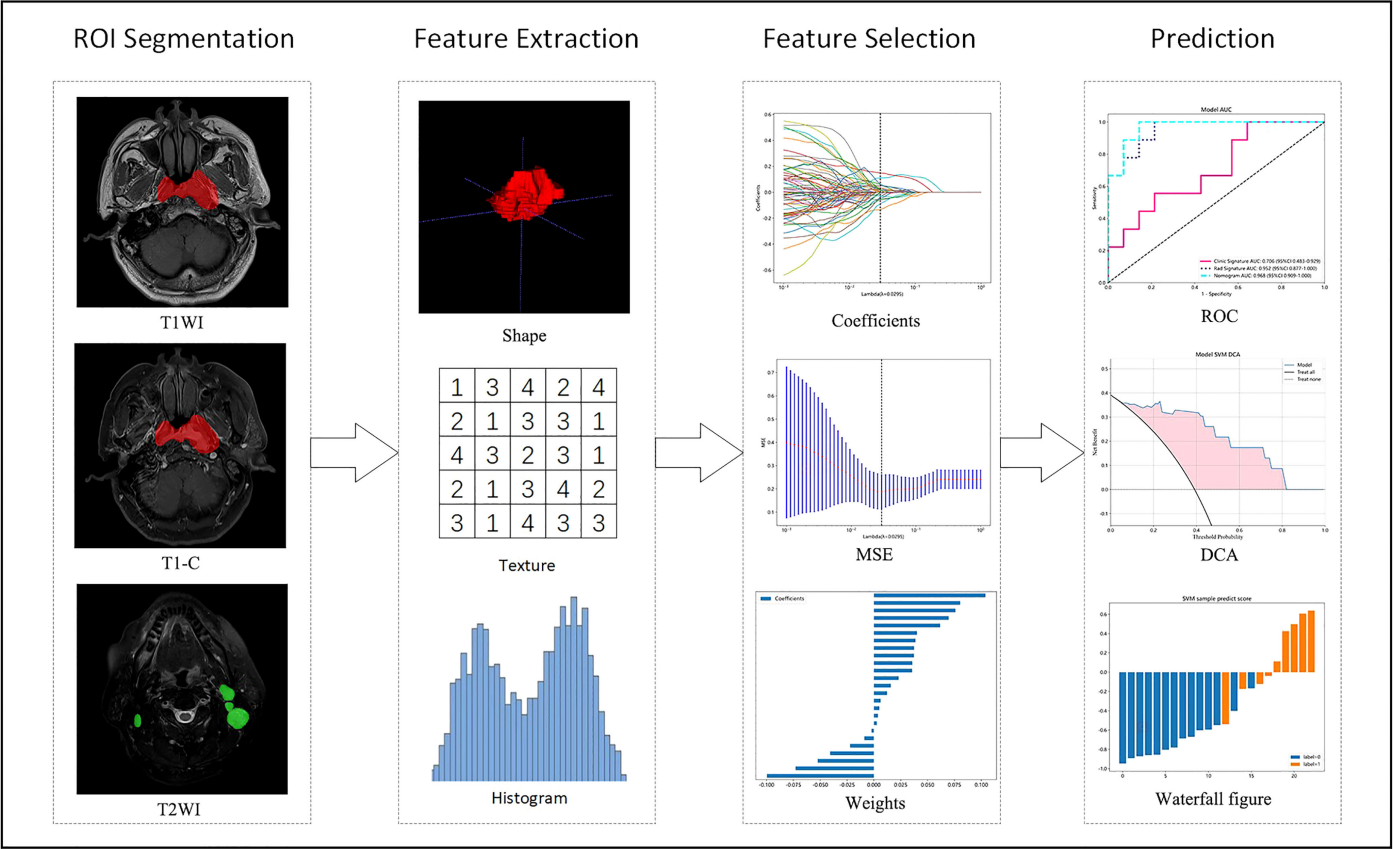
Flow chart of radiomics. T1WI, T1-weighted images; T1-C, contrast-enhanced T1-weighted images; T2WI, T2-weighted images; MSE, mean square error; ROC, receiver operating characteristic; DCA, decision curve analysis.

All patients completed image acquisition on 3.0TMR Machines. Magnetic resonance acquisition parameters are in the **Supplementary material Data S1**. DICOM format images of axial T1WI (T1-weighted images), T1-C (contrast-enhanced T1-weighted images), and T2WI (T2-weighted images) of each case were retrieved using PACS (Carestream, Ontario, Canada), and segmentations of ROI were then performed manually using ITK-SNAP (opensource software; www.itk-snap.org) by one radiation oncologists with 3 years of experience in radiotherapy for NPC. The final validation was performed by a senior radiation oncologist with 10 years of experience. They settled their differences by discussion. All radiomics features are extracted with an in-house feature analysis program implemented in Pyradiomics (http://pyradiomics.readthedocs.io). **Figure 2** illustrates the flow chart of radiomics. Due to different MRI devices, the range of pixel values of medical images varies significantly. To reduce the side-effect of pixel value outliers, we sort all the pixel values in each image and truncate the intensities to the range of 0.5 to 99.5 percentiles. Volumes of interest (VOI) are common with heterogeneous voxel spacing because of different scanners or different acquisition protocols. Such spacing refers to the physical distance between two pixels in an image. Spatial normalization is often employed to reduce the effect of voxel spacing variation. Fixed resolution (1mm × 1mm × 1mm) resampling method was used in our experiment to handle the afore mentioned problems.

### 2.6 Features selection and radiomics score construction

Clinical factors were analyzed using T-test, Mann-Whitney U tests, or χ² tests. Univariate and multivariate analyses were performed to compare the clinical characteristics between the CR group and the non-CR group. Clinical parameters with *p <*0.05 were selected to construct a clinical model.

We conducted T-test statistical test and feature screening for all radiomic features. Only the *p <*0.05 of radiomic features were kept. Pearson’s rank correlation coefficient was also used to calculate the correlation between features and one of the features with correlation coefficient greater than 0.9 between any two features is retained. We use greedy recursive deletion strategy for feature filtering, that is, the feature with the greatest redundancy in the current set is deleted each time. The least absolute shrinkage and selection operator (LASSO) regression model was used on the discovery data set for signature construction and 10-fold cross-validation was performed. After this, 25 features were finally kept. These 25 Nonzero coefficients and features were selected to establish the Rad-score with LASSO logistic regression model.

### 2.7 Model construction and evaluation

The final retained clinical features were input into classifiers such as univariate logistic regression (LR), support vector machine (SVM), K-nearest neighbor (KNN), random forest (RF), extra tree (ET), extreme gradient enhancer (XGBoost), light gradient enhancer (LightGBM) and multilayer perception (MLP) to develop the clinical model for predicting early response in LA-NPC patients. The radiomics model was constructed in the same way.

We used the logistic regression algorithm to combine clinical and radiomics features, resulting in an optimal clinical radiomic model. Next, we developed a clinical radiomics nomogram for clinical use. To evaluate the predictive ability of the three models, ROC curves were drawn for the training and validation cohorts, and the average area under the ROC curve (AUC), accuracy, sensitivity, and specificity were calculated. The clinical practicability of the models was evaluated using calibration curve and decision curve analysis (DCA).

### 2.8 Statistical analysis

The analysis was performed using various software tools, including SPSS 26 (IBM Corp., Armonk, NY, USA) and custom code written in Python v.3.7.12. Onekey v.2.2.3 platform python packages used in the analysis include scikit-learn v.1.0.2 for making machine learning algorithms, PyRadiomics v.3.0 for extracting features and statsmodels v0.13.2 is used for statistical analysis. Measurement data were expressed as mean ± standard deviation ($\bar{\text{X}}$± SD), and count data were expressed as count and percentage. Independent sample t-test, Mann-Whitney U test and ${\text{χ}}^{2}$ test were used for comparison. Significance was set at two-sided *p* < 0.05 (95% confidence interval CI).

## 3 Results

### 3.1 Clinical characteristics of the patients

Between January 2020 and January 2023, a total of 91 patients with LA-NPC were enrolled in this study. Among them, 56 patients were assigned to the CR group and the remaining 35 patients to the non-CR group based on tumor regression after treatment. The rate of CR was 61.5%, and the overall response rate (ORR) was 96.7%. **Table 1** shows the partial characteristics of all LA-NPC patients. The remaining clinical characteristics are in the **Supplementary Table S1**. Notably, PLR (*p*=0.004), nasopharyngeal tumor volume (*p*=0.008), N-cor-length (*p*=0.043), and N-axial-length (*p*=0.04) showed significant differences between the CR group and non-CR group.

**Table 1:** Characteristics of the patients.

Project	ALL	CR (n=56)	Non-CR (n=35)	*p*
Age	53.33 ± 11.54	53.86 ± 10.69	52.49 ± 12.90	0.584
Gender				0.097
Female	23 (25.27)	18 (32.14)	5 (14.29)	
Male	68 (74.73)	38 (67.86)	30 (85.71)	
Smoking				0.363
Yes	25 (27.47)	13 (23.21)	12 (34.29)	
No	66 (72.53)	43 (76.79)	23 (65.71)	
Drinking				0.669
Yes	25 (27.47)	14 (25.00)	11 (31.43)	
No	66 (72.53)	42 (75.00)	24 (68.57)	
Family history of NPC				0.585
Yes	5 (5.49)	2 (3.57)	3 (8.57)	
No	86 (94.51)	54 (96.43)	32 (91.43)	
EBV-DNA (copy/ml)				0.188
<500	68 (74.73)	45 (80.36)	23 (65.71)	
≥500	23 (25.27)	11 (19.64)	12 (34.29)	
AJCC-T				0.46
1	4 (4.40)	3 (5.36)	1 (2.86)	
2	10 (10.99)	7 (12.50)	3 (8.57)	
3	42 (46.15)	28 (50.00)	14 (40.00)	
4	35 (38.46)	18 (32.14)	17 (48.57)	
AJCC-N				0.73
1	23 (25.27)	15 (26.79)	8 (22.86)	
2	46 (50.55)	29 (51.79)	17 (48.57)	
3	22 (24.18)	12 (21.43)	10 (28.57)	
Clinical stage				0.276
III	39 (42.86)	27 (48.21)	12 (34.29)	
IVA	52 (57.14)	29 (51.79)	23 (65.71)	
Cycle of IC				1
≤2	48 (52.75)	30 (53.57)	18 (51.43)	
>2	43 (47.25)	26 (46.43)	17 (48.57)	
NLR	3.17 ± 1.94	2.87 ± 1.49	3.66 ± 2.45	0.061
MLR	0.34 ± 0.21	0.32 ± 0.12	0.38 ± 0.30	0.136
PLR	156.86 ± 64.28	141.71 ± 49.71	181.10 ± 77.18	0.004
The volume of nasopharyngeal tumor (10^3^mm^3^)	57.03 ± 43.38	47.54 ± 25.26	72.21 ± 59.69	0.008
N-cor-length (mm)	51.62 ± 24.63	47.50 ± 25.33	58.20 ± 22.25	0.043
N-axial-length (mm)	25.10 ± 12.95	22.90 ± 10.82	28.61 ± 15.29	0.04
N-total-volume (10^3^mm^3^)	26.47 ± 30.30	22.31 ± 22.38	33.11 ± 39.33	0.098

### 3.2 Radiomics feature extraction and selection

**Figure 3:**
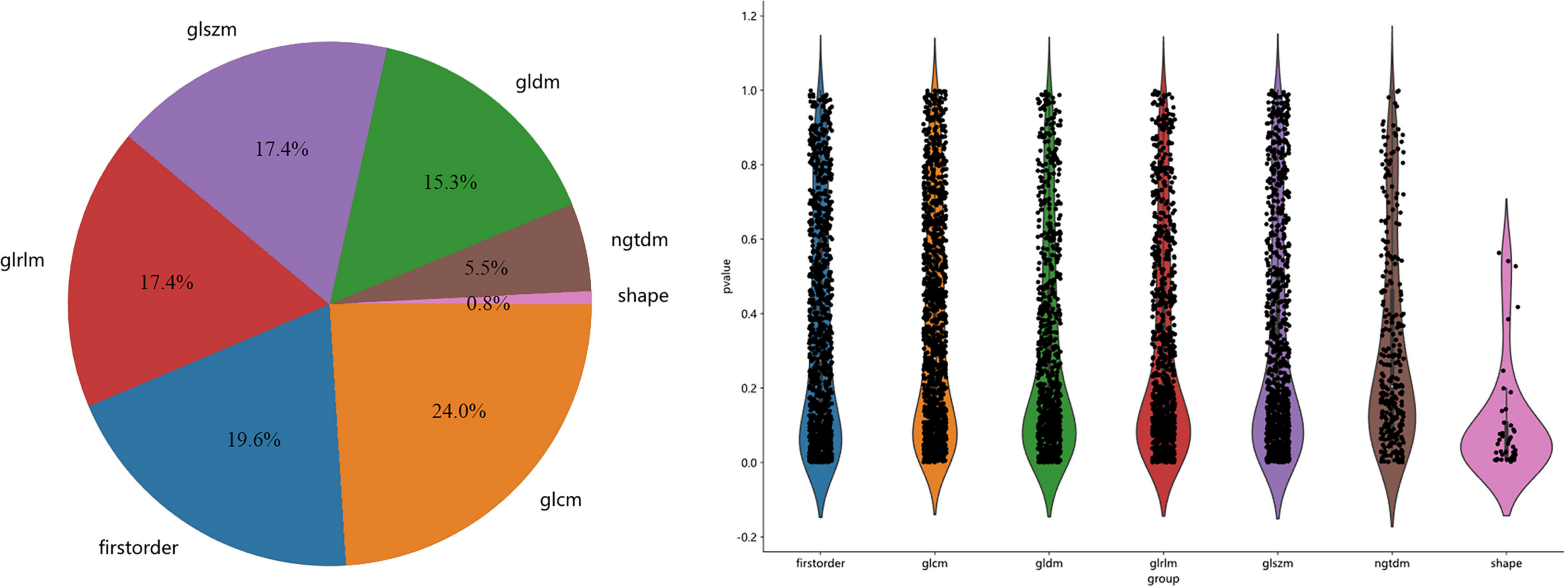
The proportion, distribution and *p* of various radiomics features.

**Figure 4:**
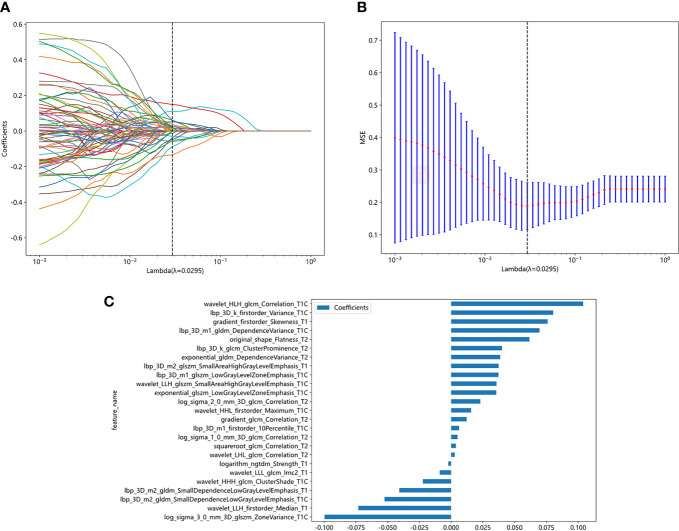
Radiomic feature selection based on LASSO algorithm. Ten-fold cross-validated coefficients **(A)** and MSE **(B)** and the histogram of the Rad score based on the selected features **(C)**.

A total of 5502 radiomics features were extracted, which included 1080 first-order features, 42 shape features, and 4380 texture features. The texture features were further divided into 1320 gray-level co-occurrence matrix (GLCM), 960 gray-level run length matrix (GLRLM), 840 gray-level dependence matrix (GLDM), 960 gray-level size zone matrix (GLSZM), and 300 neighborhood gray-tone difference matrix (NGTDM) methods. **Figure 3** illustrates the distribution and corresponding *p* of these radiomics features. Finally, the LASSO classifier selected 25 features. **Figure 4** displays the results of the 10-fold cross-validation regression, along with the final selected radiomics features and their coefficients.

### 3.3 Construction of rad-score and radiomics model

The 25 features and coefficients are fitted linearly, and the formula of Rad score is as follows:

Rad-score= 0.37486358311575735
+0.075939 * gradient_firstorder_Skewness_T1
-0.040816 * lbp_3D_m2_gldm_SmallDependenceLowGrayLevelEmphasis_T1
+0.037343 * lbp_3D_m2_glszm_SmallAreaHighGrayLevelEmphasis_T1
-0.002198 * logarithm_ngtdm_Strength_T1
-0.073031 * wavelet_LLH_firstorder_Median_T1
-0.008931 * wavelet_LLL_glcm_Imc2_T1
+0.035514 * exponential_glszm_LowGrayLevelZoneEmphasis_T1C
+0.080297 * lbp_3D_k_firstorder_Variance_T1C
+0.006255 * lbp_3D_m1_firstorder_10Percentile_T1C
+0.069564 * lbp_3D_m1_gldm_DependenceVariance_T1C
+0.037281 * lbp_3D_m1_glszm_LowGrayLevelZoneEmphasis_T1C
-0.052401 * lbp_3D_m2_gldm_SmallDependenceLowGrayLevelEmphasis_T1C
-0.099613 * log_sigma_3_0_mm_3D_glszm_ZoneVariance_T1C
-0.022222 * wavelet_HHH_glcm_ClusterShade_T1C
+0.015730 * wavelet_HHL_firstorder_Maximum_T1C
+0.103909 * wavelet_HLH_glcm_Correlation_T1C
+0.035693 * wavelet_LLH_glszm_SmallAreaHighGrayLevelEmphasis_T1C
+0.038661 * exponential_gldm_DependenceVariance_T2
+0.012215 * gradient_glcm_Correlation_T2
+0.040093 * lbp_3D_k_glcm_ClusterProminence_T2
+0.005076 * log_sigma_1_0_mm_3D_glcm_Correlation_T2
+0.023038 * log_sigma_2_0_mm_3D_glcm_Correlation_T2
+0.061645 * original_shape_Flatness_T2
+0.003769 * squareroot_glcm_Correlation_T2
+0.002816 * wavelet_LHL_glcm_Correlation_T2

**Figure 5:**
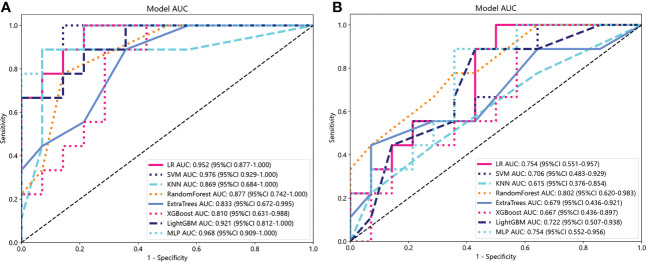
ROC analysis of different radiomics models **(A)** and clinical models **(B)** in the validation cohort.

We utilized the 25 selected radiomics features to construct a radiomics model for each classifier, and subsequently evaluated the performance of each model in both the training and validation cohorts. **Supplementary Table S2** presents statistics on the diagnostic efficacy of the radiomics models constructed by the various classifiers. Notably, the radiomics model demonstrated excellent predictive performance within the validation cohort (AUC=0.810-0.976). **Figure 5A** illustrates the ROC analysis of different radiomics models within the validation cohort.

### 3.4 Clinical model

Considering the small sample size of this study, we performed a univariate analysis of clinical parameters for all patients. The results showed that there were significant differences in PLR, N-cor-length, N-axial-length and the volume of nasopharyngeal tumor between CR group and non-CR group (*p* < 0.05). Subsequently, multivariate analysis of these four clinical parameters in the training cohort showed that PLR and nasopharyngeal tumor volume were independent predictors of early response of LA-NPC (*p*=0.017, *p*=0.012). **Table 2** showed results of multivariate analysis.

**Table 2:** Multivariate analysis.

Project	*p*	HR	95%CI	
N-cor-length	0.181	0.092	-0.021	0.205
N-axial-length	0.711	0.026	-0.091	0.143
The volume of nasopharyngeal tumor	0.012	0.124	0.043	0.204
PLR	0.017	0.122	0.039	0.206

Similar to the construction of radiomics models, PLR and nasopharyngeal tumor volume were input into each classifier to build a clinical model. Statistics on the diagnostic efficacy of the clinical models constructed by the various classifiers are presented in the **Supplementary Table S3**. RF model has the best performance, with AUC reaching 0.998 (95%CI:0.9930-1.0000) and 0.802 (95%CI:0.6202-0.9830) in training cohort and verification cohort, respectively. **Figure 5B** shows ROC analysis of different clinical models in validation cohort.

### 3.5 Establishment and validation of clinical radiomics nomogram

**Figure 6:**
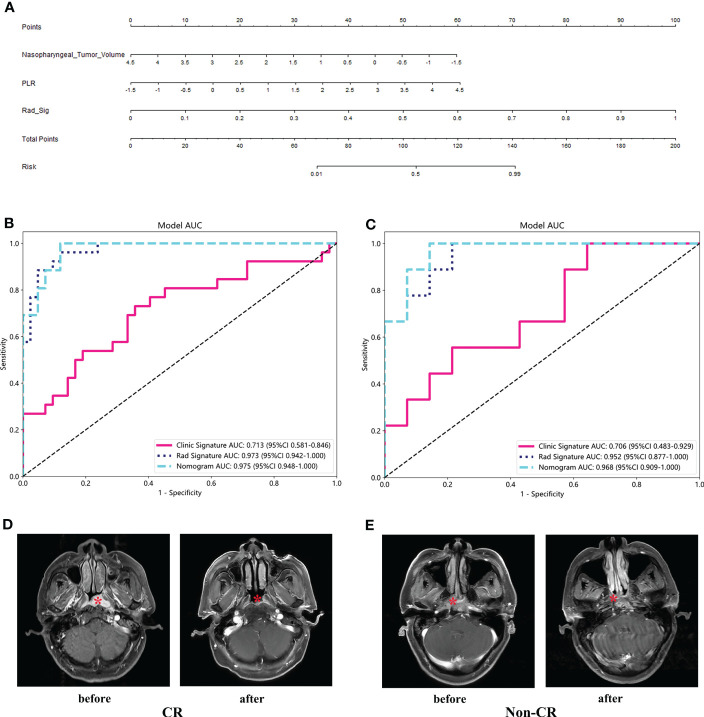
Clinical radiomics nomogram **(A)** and ROC of clinical model, radiomics model and clinical radiomics nomogram in both training cohort **(B)** and validation cohort **(C)**. T1-C images of CR and non-CR patients showing tumor response before and after treatment **(D, E)**.

Both the clinical model and the radiomics model achieved a good fit in the training cohort and the validation cohort. Logistic regression algorithm was used to combine clinical features with rad features to obtain the clinical radiomics nomogram (**Figure 6A**). The performance of the three models was compared from the aspects of accuracy, sensitivity, specificity, and AUC. All statistics are list in **Supplementary Table S4**. The AUC of clinical model, radiomics model and clinical radiomics nomogram was 0.713, 0.973, 0.975, 0.926 in the training cohort and 0.706, 0.952, 0.968 in the validation cohort, respectively (**Figures 6B, C**). The clinical radiomics nomogram provided the highest AUC in both training and validation cohort. **Figures 6D, E** show representative T1-C images of CR and non-CR patients showing tumor response before and after treatment.

### 3.6 Calibration curve and decision curve analysis

**Figure 7:**
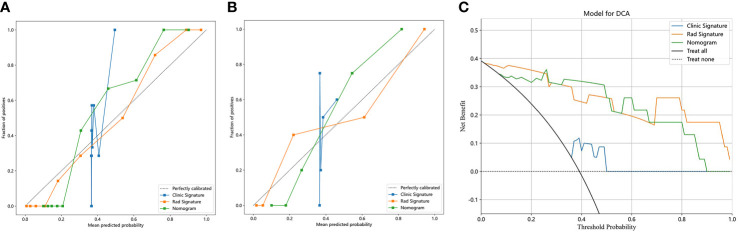
Calibration curves in the training cohort **(A)** and validation cohort **(B)**; DCA in the validation cohort **(C)**.

**Figure 7** shows the calibration curves of the three models for predicting the early response of locally advanced nasopharyngeal carcinoma in the training cohort and validation cohort. In both two cohorts, the calibration curve of the clinical radiomics nomogram and the radiomics model fit the diagonal better than the clinical model, indicating that the prediction of early curative effect of these two models for patients with locally advanced nasopharyngeal carcinoma is more realistic. **Figure 7C** shows DCA of each model in the validation cohort. The DCAs of the three models were above the two reference lines, indicating they all had clinical benefits. Among them, clinical imaging diagram and imaging model benefit more.

## 4 Discussion

In this research, it was observed that the clinical radiomics nomogram combining clinical and radiomics features had a remarkable predictive capability for determining the early response after CCRT in patients suffering from LA-NPC. It is worth mentioning that, unlike certain previous investigations, our patients were staged on the basis of the 8th edition of AJCC. Moreover, all enrolled participants underwent standard therapy of TP-induction chemotherapy in combination with platinum monotherapy concurrent chemoradiotherapy, which restrained the impact of potential confounding variables.

A number of large-scale studies have reported CR rates ranging from 82.4% to 98% after CCRT for nasopharyngeal carcinoma patients (24–27). However, our study yields a lower CR rate of 61.5%. This discrepancy can be attributed to following several factors. Firstly, we used only TP while some of the high-CR studies combined fluorouracil and TP as their IC regimen (25, 27). Secondly, more than 95% of patients in the larger studies received 3 cycles of IC, while only 47.2% of our subjects completed more than 2 cycles of IC (24, 25, 27). Those large studies had significantly more powerful treatment than that of our study. Thirdly, our study cohort had a significantly higher proportion of stage IV patients (57.1% vs 44.1% vs 30% vs 39.1%) (24–26), which may indicate a heavier tumor burden compared to other trials. Finally, different versions of the staging criteria may have influenced the assessment of tumor response. Despite the lower CR rate, our study’s ORR (96.7%) is comparable with the results of those larger studies (97.1%-98.4%) (24, 26). This implies that our treatment was just as effective in controlling the disease as the high-CR-rate studies, and our clinical data can be considered representative.

In-depth analysis was conducted in this research where multiple models were created, including the clinical model, radiomics model, and clinical radiomics nomogram. The objective was to evaluate the predictive performance of these models in determining early response post completion of CCRT in LA-NPC patients. In addition to the routinely considered clinical factors, the volume of nasopharyngeal tumor, N-cor-length, N-axial-length, and N-total volume were also taken into account as crucial parameters. The study by Zhao et al. failed to demonstrate the potential of pre-treatment nasopharyngeal tumor volume, lymph node volume and diameter in predicting IC response in NPC patients (28). However, in our study, nasopharyngeal tumor volume was an independent predictor of early response in LA-NPC patients receiving IC combined with CCRT (*p*=0.012). It suggests that the prediction of nasopharyngeal tumor volume may not be evident over a short duration. Nasopharyngeal tumor regression is more pronounced with the treatment of CCRT after IC and its prediction of response may be realized at this time. Furthermore, EBV DNA has historically been an essential indicator of NPC but its significance was not observed in this study. This might be due to the fact that our study participants were from non-endemic areas with inconsistent detection methods and low infection rates of EBV.

Wang et al. found that the radiomics model based on multiple sequences had better predictive performance than that based on a single sequence (*p <*0.05) (29). Piao et al. built prediction models based on two selected features, finding the AUC of the two features used alone (0.804, 95% CI=0.602-0.932; 0.762, 95% CI=0.556-0.905) was smaller than the combination of these two features (0.905, 95% CI=0.7240.984, *p*=0.0005) (30). The aforementioned studies indicate that both the multi-sequence model and the single-feature model exhibit superiority over their respective counterparts namely the single-sequence model and the single-feature model. Hence, our study took into consideration the construction of models based on the multi-sequence and multi-feature approach, leading to a commendable outcome. The clinical radiomics nomogram we established showed excellent AUC in both training cohort and validation cohort and performed well in accuracy, sensitivity and specificity. Our nomogram surpassed the predictive performance of both the radiomics and clinical models. Moreover, our study demonstrated the pragmatic clinical applicability of the nomogram through calibration curve and DCA. The radiomics model constructed by Xi et al. outperformed the clinical model in predicting tumor retraction after IC combined with CCRT in NPC patients (31). AUC in its training cohort and validation cohort is 0.865 and 0.819 respectively, which is lower than that of our radiomics model (AUC=0.973,0.952). The potential factors behind this difference could be the fact that they only utilized radiomics features extracted from two sequences on MRI images, whereas we utilized data extracted from three sequences. It is noteworthy that Xi et al. conducted a dynamic study of NPC radiomics by analyzing MRI images prior to and after treatment, consequently building a delta radiomics model through formulas. The delta radiomics model demonstrated superior predictive ability for both the training and validation cohorts, providing unique directions for future research in the field of NPC radiomics. Different from extracting features from T1WI, T1-C, and T2WI sequences in our study, Guo et al. utilized intravoxel incoherent motion (IVIM) parametric maps obtained from DWI images to extract radiomics features for predicting tumor treatment responses. By integrating the radiomic signature with clinical data, they built a nomogram that exhibited superior prediction ability compared to using clinical data alone (C-index, 0.929 vs 0.724, *p*<0.0001) (32). Our findings align with this result. Other studies have also corroborated that the clinical radiomics nomogram leads to superior predictive performance and accuracy compared to a simple clinical model or radiomics model (33, 34). The clinical radiomics nomogram constructed by Hu et al. to predict IC response performed better than the clinical model alone (AUC=0.81 vs 0.60, *p*=0.001). In addition, the simple radiomics model also provided a better AUC than the clinical model (0.76 vs. 0.60, *p*=0.03) (35). According to a meta-analysis, there is evidence to suggest that radiomics is a successful method for predicting the response to neoadjuvant chemotherapy in patients with NPC (36). Our paper further supports the validity of radiomics in predicting therapeutic response to NPC. The distinction lies in our ability to anticipate the therapeutic outcomes of concurrent chemoradiotherapy (CCRT), which is the prevailing therapy for patients diagnosed with LA-NPC. Given this, as compared with predicting the response to IC, it seems more valuable to forecast patients’ response to their principal treatment (CCRT) beforehand and modify treatment protocols accordingly to ensure optimal therapeutic benefits for enhancing patient survival.

Furthermore, not only MRI but also computed tomography (CT) or positron emission tomography with computed tomography (PET/CT) can be used to predict treatment response or prognosis of NPC. Hao et al. build a radiomics nomogram with 18 radiomics signatures. Patients in the high-risk group defined by this nomogram had lower 5-year disease-free survival (DFS) rate than low-risk patients (50.1% vs 87.6%, *p* < 0.0001) (37). The study conducted by Yang et al. showed great predictive performance of CT-based model using deep learning features for identification of responders and non-responders to IC in NPC (38). Our study chose MRI to build radiomics model and nomogram as MRI has become the preferred imaging method for NPC due to its low invasiveness, cost-effectiveness, and high accuracy. In the future, MRI, CT, PET/CT and other different types of images may be combined to serve the diagnosis and treatment of NPC.

In the present investigation, we developed a nomogram that incorporates clinical and radiomics features that effectively forecast the initial response to CCRT in patients diagnosed with LA-NPC. Nevertheless, it is pertinent to underscore that there exist certain limitations within this study. Firstly, this research was a retrospective study conducted in a single center which failed to undergo external validation and the sample size was limited. Secondly, since long-term survival follow-up data was not available, we could not undertake a thorough analysis of PFS and OS of LA-NPC patients. In the future, we will intend to expand the data and further focus on patient survival rates and overall prognosis.

## 5 Conclusion

The utilization of an MRI-based clinical radiomics nomogram has demonstrated superior ability to predict early response for LA-NPC patients as compared to simplistic clinical or radiomics models. This nomogram has the capacity to identify patients who fail to attain CR following CCRT at earlier stages, thereby facilitating timely intervention treatment or personalized therapy can be carried out to improve the patient’s survival and prognosis.

## Data availability statement

The raw data supporting the conclusions of this article will be made available by the authors, without undue reservation.

## Ethics statement

The study was approved by the local scientific research ethics committee, and informed consent was waived because it was a retrospective study. The study process was in accordance with the Declaration of Helsinki Ethics statement.

## Author contributions

MW, WX and YF contributed equally to this article and share first authorship. MW, WX and YF collected the clinical data. YL, JY and LQ collected MRI images. YZ, GC and YCh completed the data sorting and induction. MW finished segmentation of the MRI images. MW, WX and YF analyzed the data and wrote the manuscript. YCa validated ROI. XS and SZ provided study supervision, article revision and project funding. All authors contributed to the article and approved the submitted version.
