# Supplementary material for: MRI-based clinical radiomics nomogram may predict the early response after concurrent chemoradiotherapy in locally advanced nasopharyngeal carcinoma
Source: Front Oncol. 2023 May 15;13:1192953. doi: 10.3389/fonc.2023.1192953 (PMC10225671; doi:10.3389/fonc.2023.1192953)
Supplement: Supplementary file 1 [file Presentation_1.pdf]

Supplementary Material

MRI-based clinical radiomics nomogram may predict the early response after concurrent chemoradiotherapy in locally advanced nasopharyngeal carcinoma

Mengxing Wu[1,2] †, Weilin Xu[1] †, Yinjiao Fei[1] †, Yurong Li[1,2], Jinling Yuan[1,2]， Lei Qiu[1,2], Yumeng Zhang[3], Guanhua Chen[4], Yu Cheng[5], Yuandong Cao [1]*, Xinchen Sun[1,2]* and Shu Zhou[1]*

*** Correspondence:** Corresponding Author:
Shu Zhou
zhoushu164086035@126.com

Xinchen Sun
sunxc210@126.com

Yuangdong Cao
yuandongcao@163.com

1 Data S1 Magnetic resonance acquisition parameters:

（1） GE 750W 3.0T MR：

T1WI：TR=825ms，TE=11ms，FoV read=240mm，FoV phase=100%，Slice thickness=5mm，Averages=2；

T1-C：TR =165.1ms，TE=9ms，FoV read=240mm，FoV phase=100%，Slice thickness =5mm，Averages=2；

T2WI：TR =3236ms，TE =85ms，FoV read=240mm，FoV phase=100%，Slice thickness=5mm，Averages=2；

（2）Philips Ingenia 3.0T:

T1WI：TR =650ms，TE =18ms，FoV read=250mm，FoV phase=100%，Slice thickness=5mm，Averages=2；

T1-C：TR =650ms，TE =9ms，FoV read=250mm，FoV phase=100%，Slice thickness=5mm，Averages=2；

T2WI：TR =3500ms，TE =90ms，FoV read=250mm，FoV phase=100%，Slice thickness=5mm，Averages=2；

2 Tables

2.1 Table S1 Characteristics of the patients.

Project	ALL	CR(n=56)	Non-CR(n=35)	*p*
Dose of Platinum in CCRT（mg）				0.628
＜240	40(43.96)	23(41.07)	17(48.57)	
≥240	51(56.04)	33(58.93)	18(51.43)	
BMI	23.99±3.56	24.21±3.74	23.64±3.28	0.463
WBC (10^9/L)	6.70±1.87	6.58±1.74	6.90±2.07	0.436
Lymphocyte(10^9/L)	1.58±0.46	1.65±0.47	1.48±0.41	0.079
Monocyte(10^9/L)	0.50±0.19	0.49±0.17	0.50±0.21	0.889
Neutrophils(10^9/L)	4.48±1.68	4.29±1.56	4.78±1.85	0.175
PLT (10^9/L)	231.59±74.68	221.80±74.61	247.26±73.15	0.114
ALP(U/L)	85.49±22.93	85.27±20.81	85.86±26.29	0.906
LDH(U/L)	198.57±59.47	192.05±38.05	209.00±82.69	0.188
Alb (g/L)	41.01±3.89	41.31±3.07	40.53±4.95	0.357
D-Dimer(mg/L)	0.39±0.64	0.32±0.30	0.51±0.95	0.166

2.2 Table S2 Statistics on the diagnostic efficacy of the radiomics models constructed by the various classifiers.

Model	Cohort	Accuracy	Sensitivity	Specificity	AUC	95% CI
LR	Training	0.912	0.962	0.881	0.973	0.942 - 1.000

	Validation	0.870	1.000	0.786	0.952	0.877 - 1.000
SVM	Training	0.956	1.000	0.929	0.993	0.981 - 1.000
	Validation	0.913	1.000	0.857	0.976	0.929 - 1.000
KNN	Training	0.882	0.923	0.857	0.955	0.912 - 0.998
	Validation	0.913	0.889	0.929	0.869	0.684 - 1.000
RF	Training	1.000	1.000	1.000	1.000	nan - nan
	Validation	0.826	0.778	0.857	0.877	0.742 - 1.000
ET	Training	1.000	1.000	1.000	1.000	nan - nan
	Validation	0.739	0.889	0.643	0.833	0.672 - 0.995
XGBoost	Training	1.000	1.000	1.000	1.000	nan - nan
	Validation	0.783	0.889	0.714	0.81	0.631 - 0.988
LightGBM	Training	0.838	0.962	0.762	0.924	0.865 - 0.983
	Validation	0.826	0.889	0.786	0.921	0.812 - 1.000
MLP	Training	0.926	0.962	0.905	0.973	0.936 - 1.000
	Validation	0.913	0.889	0.929	0.968	0.909 - 1.000

2.3 Table S3 Statistics on the diagnostic efficacy of the clinical models constructed by the various classifiers.

Model	Cohort	Accuracy	Sensitivity	Specificity	AUC	95% CI
LR	Training	0.706	0.538	0.810	0.663	0.525 - 0.802
	Validation	0.696	1.000	0.500	0.754	0.551 - 0.957
SVM	Training	0.676	0.731	0.643	0.713	0.581 - 0.846

	Validation	0.609	1.000	0.357	0.706	0.483 - 0.929
KNN	Training	0.662	0.731	0.634	0.697	0.578 - 0.816
	Validation	0.609	0.444	0.769	0.615	0.376 - 0.854
RF	Training	0.971	0.962	0.976	0.998	0.993 - 1.000
	Validation	0.696	0.778	0.643	0.802	0.620 - 0.983
ET	Training	1.000	1.000	1.000	1.000	0.000 - 1.000
	Validation	0.739	0.444	0.929	0.679	0.436 - 0.921
XGBoost	Training	0.926	1.000	0.881	0.989	0.973 - 1.000
	Validation	0.652	1.000	0.462	0.667	0.436 - 0.897
LightGBM	Training	0.662	0.615	0.707	0.693	0.561 - 0.825
	Validation	0.696	0.889	0.615	0.722	0.507 - 0.938
MLP	Training	0.676	0.615	0.714	0.684	0.549 - 0.819
	Validation	0.739	0.889	0.643	0.754	0.552 - 0.956

2.4 Table S4 Statistics on the diagnostic efficacy of three models in the training and validation groups.

	Training			Validation		
	Clinical	Radiomics	Nomogram	Clinical	Radiomics	Nomogram
Accuracy	0.676	0.912	0.926	0.609	0.870	0.913
Sensitivity	0.731	0.962	1.000	1.000	1.000	1.000
Specificity	0.643	0.881	0.881	0.357	0.786	0.857
AUC	0.713	0.973	0.975	0.706	0.952	0.968

95% CI	0.581 - 0.846	0.942 - 1.000	0.948- 1.000	0.483 - 0.929	0.877 - 1.000	0.910 - 1.000
